# Content categorization for memory retrieval: A method for evaluating design performance

**DOI:** 10.1371/journal.pone.0280459

**Published:** 2023-01-19

**Authors:** Danni Shen, Xuelin Yao, Defu Bao, Yuxiang Yu

**Affiliations:** School of Art and Design, Zhejiang Sci-Tech University, Hangzhou, Zhejiang, China; Universiti Malaysia Pahang, MALAYSIA

## Abstract

Designers search for memories and retrieve appropriate mental information during design brainstorming. The specific contents of retrieved memories can serve as stimuli for new ideas, or act as barriers to innovation. These contents can be divided into different categories, which are reflected in designers’ creativities, and derived from individual lives and design experiences. Appropriate categorization of retrieved memory exemplars remains a fundamental research issue. This study tentatively divided retrieved memory exemplars into eight categories from brainstorming on the topic of library desk and chair design. A verification questionnaire was performed and validated the accuracy of categorization. The categorization result could be applied to design education in terms of understanding students’ design performances and capabilities.

## Introduction

Memory retrieval is referred to the subsequent re-accessing of events or information from the past, which have been previously encoded and stored in the brain [1]. Research on memory retrieval has a long history in psychology [2, 3], which mainly focused on the mechanisms and extraction methods through psychology experiments. In the field of design, memory retrieval is represented as a cognitive process in order to study its impact on idea generation [4–7].

Memory retrieval plays an important role in creative activities. Designers’ creative activities are based on their pre-stored experiences in the brain, which are accumulated from discovering and solving daily-life problems. The information stored in long-term memory [8], provides the stimulus for future design creativities. Through memory retrieval, association, transformation and other complex brain activities, the stored information can be converted into new ideas [5], or used subconsciously to influence designers [9]. Although the information cannot be directly transformed into new ideas, the idea generation necessarily involves the processes of memory retrieval [8, 10] and relies on the content of retrieved memory. It is difficult for designers to generate new ideas without certain memory. Previously stored knowledge must be used, as ideas cannot be generated ex nihilo [11].

The impacts of memory retrieval on idea generation is various and depend not only on the external conditions of experience acquisition process [12, 13], but also on the retrieved content under conditions of creative activities and individual thinking ability [14–17]. On one hand, designers with different experience levels may produce various ideas from the same category of the content. On the other hand, different design issues may produce different categories of retrieved memory, which in turn generate different ideas.

The content of memory retrieval is accumulated from lives and design experiences [18], and are the primary factors resulting in significant differences between experts and novices [19, 20]. Compared with novices, experts with extensive experiences can extract more information from the long-term memory [21] and also have a broader set of mental models stored in memory to form the initial solution space [22]. This implies that they can draw upon more sophisticated mental models based on their accumulated design knowledge and expertise to trigger stimuli more effectively, resulting in higher-quality designs.

While addressing different design issues, designers will extract different categories of content from memory. For problem-solving design issues, designers may find defective parts, e.g., noise from dragging chairs, in the current mode of operation through personal experiences or user behavior research, and further analyze the source of problem [23]. To find solutions, designers would recall and reference solutions from analogous designs to create innovative solutions for problem [24–27]. Relevant information may be linked to the mechanical structure, or a specific problem encountered in the interaction with product. Designers can apply the structure, function, and application from other areas to the design problem at hand via logical reasoning. For appearance design problems, designers can develop a novel product appearance by retrieving the design similar to the design topic and using visual reasoning. Designers can also create unusual designs by extracting key elements from objects distant from the design topic. To this end, designers rely on their imagination and integration abilities to analyze and compare the information. In this manner, the retrieved memory can act as a stimulus to promote designers’ abilities on generating innovative ideas.

In conclusion, understanding different categories of retrieved memory can help designers with different experience levels to conduct more targeted and efficient brainstorming activities when facing various design problems, and then promote the generation of creative ideas. As to the specific categories of retrieved memory, some researchers have implicitly focused on the certain categories in the study of memory retrieval and design fixation. Similar designs or existing products may affect designers’ thinking [23, 28–31]. Design ideas combined with far-related concepts tend to yield more innovative results [32–34]. Although these investigations involve particular categories in an implicit way, these categories have not been studied using the same categorization system. There may also be categories of retrieved memory not yet documented. The work, systematically categorizing existing categories and finding new categories under the same standards, is the fundamental basis of the above research issues.

This study proposed a categorization method for retrieved memory, which will provide a standard category reference for designers to analyze and compare information in design creativities. Study 1 proposed the categorization method and conducted an experiment to show the categorization process. Study 2 verified the accuracy and acceptability of the categorization method. And in Study 3, the categorization results were preliminarily applied to the analysis of the design process.

## Study 1

Study 1 proposed a categorization method, obtained memory retrieval sentences through a brainstorming experiment, and set categorization criteria to make categorization questionnaire. The categories of memory retrieval were obtained by analyzing the questionnaire scores through hierarchical cluster analysis.

### Participants

In the brainstorming experiment, participants were required to have good design skills. Fourteen post-graduate students (six women, eight men) aged 23–26 majoring in industrial design were invited. They had systematically learned how to design for at least four years. In the categorization questionnaire, a total of 31 college students (aged 21–27; 13 women, 18 men) were invited. They all had normal language comprehension and were not involved in the brainstorming experiment. The study was approved by the School of Art and Design, Zhejiang Sci-Tech University.

### Methods

#### Brainstorming and design topic

Brainstorming is a method in which participants can think freely and explore new ideas and solutions to problems based on a given topic without constraints [35]. One of the four famous principles of brainstorming is to strive for quantity [35]. In order to obtain as much memory retrieval content as possible, a typical and less restrictive design topic–library desk and chair design–was chosen, which is also a representative of typical content in design education and covers a wide range of design issues [36]. Although the requirements for participants in this topic were relatively vague, previous studies have proved that vague goals may strengthen observed the links between quality and quantity during idea generation by making the variation of observed ideas be more random [37]. In many design studies using brainstorming, the design topics were also relatively vague, such as office exercise equipment [38] and small electro-mechanical consumer products [39].

#### Categorization criteria

To understand the meaning of a certain sentence, we started the categorization from Semantics and elements of Meaning for categorization criteria. Ogden et al. [40] proposed 22 kinds of definitions of Meaning, which were aggregated into Groups A, B and C. Li [41] believed that Group A was generalized as Referent from the view of natural characteristic of things; Group B was generalized as Symbol and expressed the relationship between one word with other words; and Group C was from the point of Users. These groups composed the three elements of Meaning. In addition, Leech [42] developed seven categories of Meaning, e.g., affective and thematic meaning. According to the form of quadruple to describe “opinion” proposed by Kim & Hovy [43], we transformed it to the categorization criteria of memory retrieval as [Topic, Type, Property, and Valence], specifically.

- Distance to the topic (far–close): Some of the retrieved memories are far from the given design topic, while some are close. This categorization criterion was used to evaluate the distance between the sentence object and the design topic (library desk and chair design).
- Description type (object–event): Referent is an important element of Meaning. From the aspect of memory retrieval, we called the Referent as Description Type. This categorization criterion was used to evaluate whether a sentence was describing an object that the designer has ever seen or an event experienced.
- Description property (objective–subjective): While describing an object/event, designers may be affected by personal subjective factors. For example, the sentence “The seats of Zijingang Library are made of hardwood” biased towards objective, while the sentence “The seats of Zijingang Library made of hardwood make me comfortable/uncomfortable” biased towards subjective as comfort is associated with personal preference. This categorization criterion was used to evaluate objectivity and subjectivity in sentence description.
- Valence (negative–positive): The affective meaning is one of the seven categories of Meaning. This categorization criterion was used to evaluate the degree of negation or positivity of a sentence description. For example, the sentence “The seats of Zijingang Library made of hardwood make me comfortable (uncomfortable)” biased towards positive (negative).

#### Categorization questionnaire and hierarchical clustering

Based on the four categorization criteria, a semantic differential scale questionnaire was constructed for the collected sentences from the brainstorming. The settings of categorization questionnaire are shown in Table 1. The questionnaire adopts 7-point scale, where numbers represent degree: 1 and 7 means Extremely, 2 and 6 means Quite, 3 and 5 means Slightly, and 4 means Neutral. And the numbers 1, 2 and 3 mean biasing to the left word, i.e., close, object, objective or negative, while numbers 5, 6 and 7 mean biasing to the right word, i.e., far, event, subjective or positive. To eliminate the error caused by the sequence of questionnaire, the sentence order of each questionnaire was randomized.

**Table 1:** Examples of categorization questionnaire.

42. It will be very noisy when study in the library if some people are discussing aside.							
Distance to the topic (close–far)	1	2	3	4	5	6	7
Description type (object–event)	1	2	3	4	5	6	7
Description property (objective–subjective)	1	2	3	4	5	6	7
Valence (negative–positive)	1	2	3	4	5	6	7

Hierarchical clustering was used to process the data from the categorization questionnaire. It is a method to build a hierarchy of clusters in data mining and statistics[44]. We planned to use an agglomerative strategy where each object initially represents a cluster of its own and then the clusters are merged according to a certain metric.

### Procedure

#### Individual brainstorming

In the brainstorming experiment, the participants were required to think aloud [45], i.e., to verbally speak out all the thinking process in their minds. The brainstorming was conducted in a closed room. We provided the participants paper, pencils and erasers. The design process of each participant was recorded by a camera, and the data were collected in video and audio formats.

Before the formal brainstorming, each participant was outlined the requirements, process and design topic of preliminary brainstorming. The preliminary brainstorming focused on a medication cup and lasted for 10 minutes to allow participants to adapt to the method of thinking aloud. After 5 min break, the participants started formal 35-minute brainstorming. To ensure the integrity and reliability of data, each participant was interviewed after the brainstorming, during which they explained the design thought related to each idea and supplemented the content of memory retrieval based on camera records.

#### Sentence collation and processing

The recorded video and audio data needed to be converted into textual form for further study. The textual data mainly contained three kinds of contents—retrieved memory, design steps and irrelevant sentences. Two Ph.D. candidates majoring in design screened and recorded the retrieved memory sentences, respectively. The record process was based on the following rules: Sentences describing one specific item were counted as one sentence, and sentences describing the same item at different times were counted as one sentence. A total of 172 sentences were recorded (S1 Data). The Cohen’s Kappa consistency check of the two recorders was 0.686, p<0.001, good consistency.

Some sentences from different participants were found to be evidently similar on the aspect of expressions, e.g., the same meaning or descriptions of the same topic. The similar sentences were combined without affecting the meaning of sentence descriptions. For instance, participant A thought “the library has a study room and reading room”, while participant B thought “the library could be divided into a self-study area and discussion area”. Since both sentences shared the same meaning of dividing the library into two functional blocks, they were combined as “the library has room for study, reading and discussion”. The combination work was jointly completed by the two aforementioned Ph.D. candidates, through which a total of 152 sentences remained (S2 Data).

#### Categorization questionnaire survey

The above sentences were used for the categorization questionnaires. Before the categorization questionnaire survey, the participants were required to read a questionnaire lead carefully, including interpretation of the four criteria, score settings, and questionnaire process. Each participant was asked to rest for 10 min after completing half the questionnaire to eliminate fatigue effects.

Hierarchical clustering method was adopted to deal with the data from the categorization questionnaires. The raw data were the scores of 152 sentences on 4 categorization criteria (S3 Data), and the metric was Pearson correlation which indicated the similarity of 152 sentences. However, the hierarchical clustering is an unsupervised learning method, and once a group of objects is merged, the process at the next step will operate on the newly generated clusters. It will neither undo what was done previously, nor perform object swapping between clusters [46]. It means that errors may be accumulated, and thus lead to low-quality or even wrong clusters. To avoid this case, we obtained clusters at initial layers and then adopt manual intervention.

**Fig 1:**
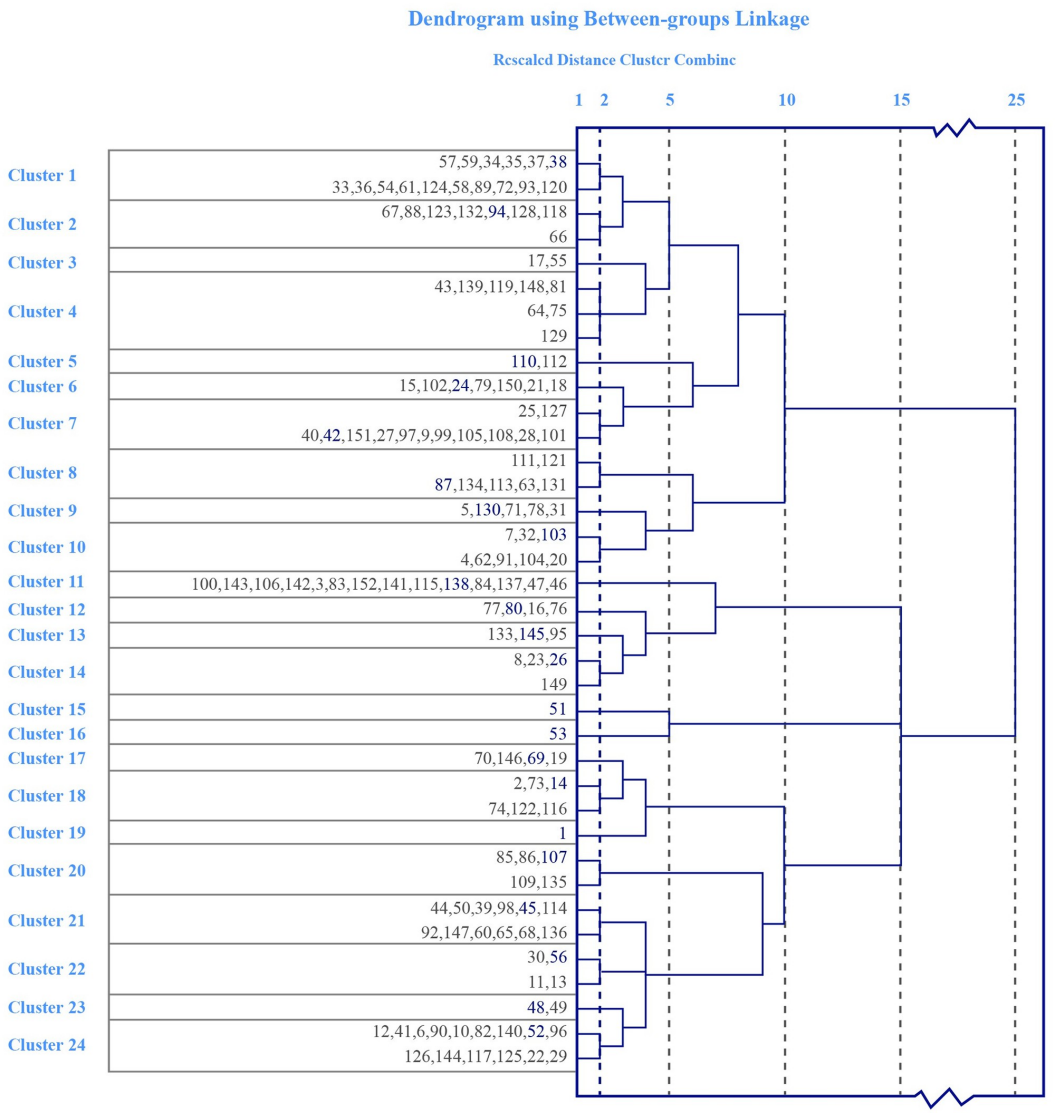
Results of hierarchical cluster analysis of 152 sentences and 24 clusters.

The clustering procedure was realized via SPSS, and its results are showed in Fig 1. The middle and the top numbers represent the number of sentences and the number of hierarchies, respectively. The first layer of 152 sentences is grouped into 37 clusters, which was still too large for artificial categorization analysis. Therefore, we cut the tree at the second layer and obtained 24 clusters for tractability, which are segmented by a black dash line in Fig 1. The variance analysis of both layers shows statistical significance (p <0.01) for each categorization criterion, which means clustering is reasonable.

#### Artificial categorization

A focus group [47] consisted of two Ph.D. candidates and four postgraduates was convened to discuss the practical implications of the 24 clusters. Selecting a typical sentence from each cluster as a reference for category naming. After clustering, the sentences in the same cluster have similar scores, which constitutes unique characteristics of each cluster itself. Given any cluster, we use Pearson coefficient to define the distance between any two sentences. The smaller distance of two sentences means more similar. Intuitively, for any given cluster, there exists a sentence that the summation of distances to other sentences is the smallest. This sentence is defined as the center of cluster. The focus group selected the center sentence as typical sentence of cluster, which are marked with blue font in Fig 1 and listed in S2 Data.

To make the logic of naming more understandable, we take Cluster 13 as example. As shown in Table 2, Cluster 13 tends extremely to Positive, quite to Close and Object, and a little to Subjective. The typical sentence is “The chairs in a high-speed rail or airplane first class are very comfortable”. Synthesize these characteristics, the focus group named the Cluster 13 as “Other types of good desk and chair design”.

**Table 2:** Summary of Cluster 13.

Cluster 13	Distance to topic	Description type	Description property	Valence
Scores on criteria	2.72	2.38	4.84	5.41
Typical sentence	The chairs in a high-speed rail or airplane first class are very comfortable.			

Given the 24 clusters and based on the unique characteristics of each cluster as well as its practical meaning revealed via its name, the focus group performed artificial categorization, where 24 clusters were merged into 8 categories. For example, Clusters 1, 4 and 15 described the desk and chair designs from existing library, no matter these designs were of good or bad quality. Therefore, the three clusters were aggregated into Category 1 named as Congeneric product design. Besides, 109-cluster 22 and 135-cluster 22 simply expressed that the designer had seen foreign library desk and chair designs before, rather than described the designs in detail, which made the participant consider these two sentences as events in the categorization questionnaire. The focus group believed that the designer wanted to emphasize the specific content of foreign library desk and chair design. Hence, the two sentences were categorized into this category.

### Results

**Fig 2:**
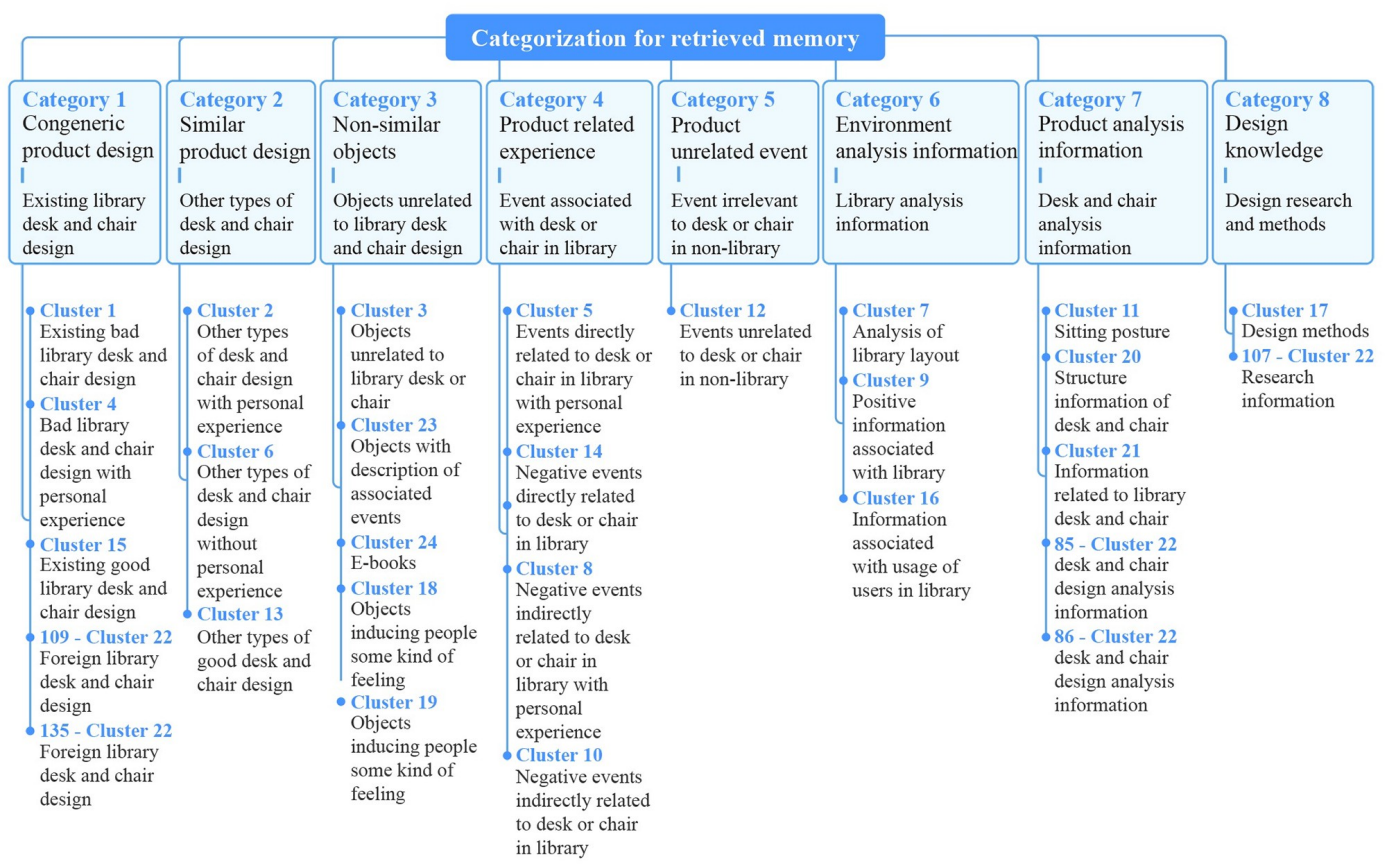
Artificial categorization process of retrieved memory based on practical implications.

The whole 24 clusters and artificial categorization processes are shown in Fig 2. The interpretations of each category are as follows.

- Category 1 Congeneric product design: The category includes objects of the same kind. In terms of this design topic, the category contains design cases with the object of desk or chair, and the usage situation is the library. Therefore, the example category under the design topic is existing library desk and chair design.
- Category 2 Similar product design: The category refers to the design of objects similar to the design object. With regard to this design topic, the category contains designs where the design object is a desk or chair, and the usage situation is not a library, such as aircraft seat. The example category under the design topic is other types of desk and chair design.
- Category 3 Non-similar objects: The category contains all objects with the exception of congeneric or similar objects. In the aspect of the design topic, the example category is objects unrelated to library desk and chair design, e.g., plasticine.
- Category 4 Product-related experience: The category refers to the events happening when designers interact with the design object. These events tend to be negative. In terms of design topic, the example category is event associated with desk or chair in the library.
- Category 5 Product unrelated event: The category refers to events unrelated to the design object. With regard to design topic, this category incorporates the events irrelevant to desks or chairs in a non-library setting, which is the example of this category.
- Category 6 Environment analysis information: The category refers to analysis information about the usage situation of the design object based on design research and methods. With respect to the design topic, this category mainly refers to library analysis information, which is the example of this category.
- Category 7 Product analysis information: This category refers to analysis information about the design object based on design research and methods mastered by designers, such as structural features and use-patterns. As for the design topic, this category describes desk and chair analysis information, which is the example of this category.
- Category 8 Design knowledge: This category is a large body of knowledge that designers call upon and use during the design processes to match the ever-increasing complexity of design problems [48]. However, the brainstorming time limited the designers’ deeper thinking on the design topic. The quantity of Category 8 was very small. But this category did exist and was difficult to be joined into other categories. Therefore, the focus group retained this category. With respect to this design topic, this category incorporates design research and design methods.

## Study 2

To verify the accuracy and acceptability of the categorization, Study 2 conducted a verification questionnaire survey.

### Participants

Twenty-two college students (21–25 years; 10 women and 12 men) were invited to participant in the verification questionnaire. The requirement for participants were the same with Categorization Questionnaire, who were assumed to have normal language comprehension. These participants did not take part in the previous categorization questionnaire, so that all of them were not influenced by prior procedure.

### Methods

The twenty-four typical sentences were selected from the second layer of hierarchical clustering for the verification questionnaire. Since cluster 22 of the second layer had been split, the five sentences of cluster 22 were also added to the verification set. One of the five sentences had been included in the 24 typical sentences. Finally, a list of 28 sentences were selected for questionnaire verification (S2 Data).

In the questionnaire, each sentence corresponds two choices, i.e., categories and example categories (S1 Table). Options selected for each choice include all categories and example categories under the design topic of library desk and chair design as well as an “other” option. If there are many participants choose the option “other”, it means that the above categorization is not comprehensive enough.

Correspondence analysis (CA) [49] is used to analyze and verify the results of the questionnaire. CA is multivariate statistical technique and usually applies to categorical variables, which provides a means of displaying or summarizing categorical data in dimensional and graphical forms. Given a set of description from individuals on several categorical variables, CA creates orthogonal components to present the descripted variables with possibly lower orthogonal dimensions [50].

### Results

The result of CA directly generated by SPSS is shown in Table 3. The proportion of inertia represents the percentage of each dimension. The first inertia explains 17% of the total information; the second inertia explains 16.3% of the total information and the cumulative explanation is 33.3%. Although the eighth inertia explains 3.3%, it is not trivial. The cumulative value of the eight-dimensional inertia is 1. The eight example-categories/categories cannot be further merged to fewer dimensions, indicating that only these eight dimensions can fully represent the information. Therefore, the eight example-categories and the eight categories are sufficient to cover the content of memory retrieval.

**Table 3:** Summary of correspondence analysis.

Dimension	Inertia	Chi Square	Sig.	Proportion of Inertia	
				Accounted for	Cumulative
1	.840			.170	.170
2	.806			.163	.333
3	.767			.155	.488
4	.707			.143	.631
5	.647			.131	.761
6	.587			.119	.880
7	.429			.087	.967
8	.165			.033	1.000
Total	4.947	3047.634	.000^a^	1.000	1.000

^a^. 64 degrees of freedom

**Fig 3:**
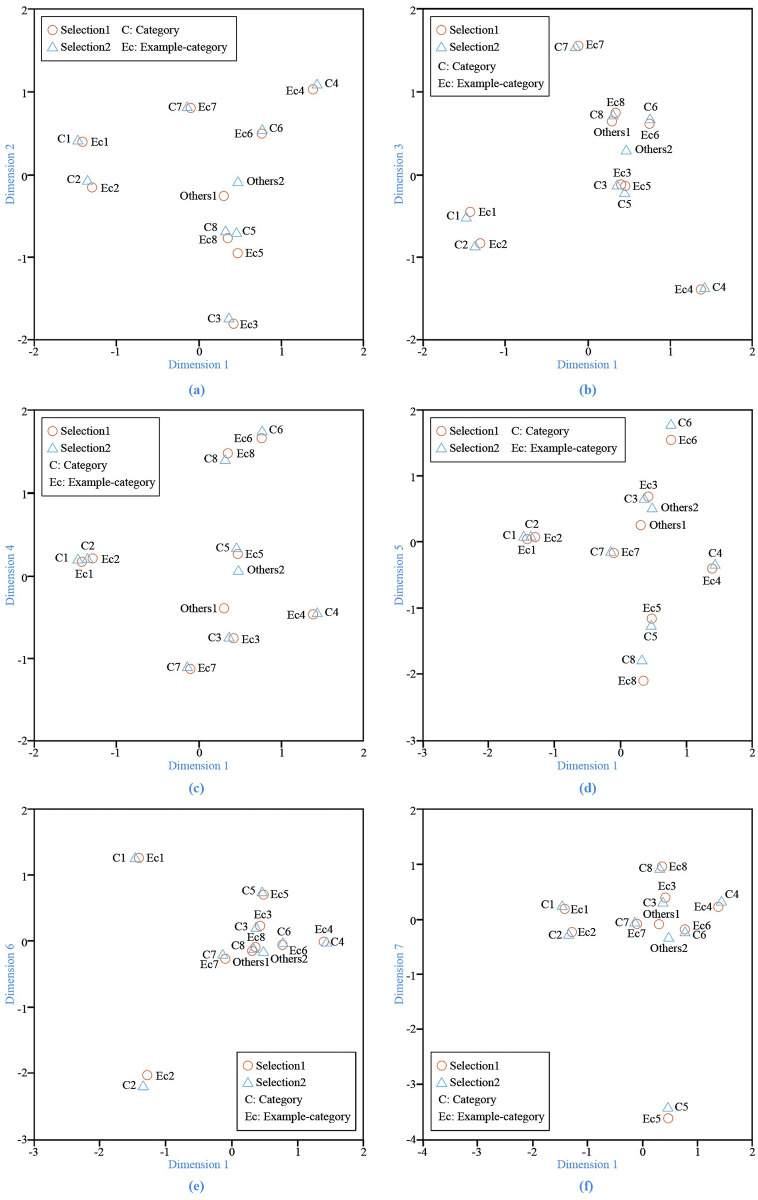
Correspondence analysis scatterplot.

On the other hand, Fig 3 shows the correspondence relationship between example-categories and categories in different dimensions. Selection 1 represents category selection, and Selection 2 represents example-category selection. It can be observed that all of the example-categories except Example-category 5 fit well regarding to the corresponding categories by considering Dimensions 1 and 2. The correspondence between Example-category 5 and Category 5 can be considered from other dimensions, e.g., Dimensions 1 and 5 as shown in Fig 3(d).

## Study 3

Study 3 quantitatively analyzed the sentences of 14 participants according to the eight categories to observe their memory retrieval and inspiration in the brainstorming experiment.

### Methods

In addition to the retrieved memory, the sentences obtained from brainstorming also included descriptions of design ideas. In other words, which ideas were inspired by the retrieved memories could be analyzed. In this study, the retrieved memory that stimulates idea generation is called inspiration. The 172 raw sentences of the 14 participants were statistically analyzed according to the 8 categories.

### Results

Table 4 shows the distribution of sentences and inspirations for 14 participants in 8 categories. To the left of “/” is the number of inspirations, and the right is the number of sentences. For example, for the first participant in category 1, the number of inspirations is 4, and the number of sentences is 7. No numbers indicates that the participant did not retrieve any sentences on the category. The number of inspirations divided by the number of sentences is used as the creative conversion ratio of memory retrieval. A high conversion ratio means that this category of memory is efficient at stimulating ideas.

**Table 4:** Distribution of sentences and inspirations for 14 participants in 8 categories.

	Category								
Participant	1	2	3	4	5	6	7	8	Total
1	4/7	5/6	1/1	5/5	0/1	1/4	1/1	/	17/25
2	1/1	2/2	/	4/9	/	1/3	/	/	8/15
3	/	0/2	/	2/5	/	1/1	/	/	3/8
4	0/2	1/2	4/4	/	/	0/1	/	1/2	6/11
5	/	1/1	/	4/5	0/1	/	0/2	/	5/9
6	0/3	2/2	/	2/2	/	2/2	3/4	/	9/13
7	0/1	0/1	2/2	2/3	/	/	1/3	/	5/10
8	1/2	2/2	2/2	2/2	/	0/1	2/4	/	9/13
9	/	2/2	2/2	1/2	/	/	1/2	/	6/8
10	2/2	/	4/4	4/5	/	/	0/1	1/1	11/13
11	0/2	1/2	1/1	0/1	/	2/3	1/3	/	5/12
12	0/1	2/2	1/1	2/5	/	0/1	3/5	/	8/15
13	0/2	0/1	4/5	1/1	/	0/1	/	1/1	6/11
14	0/3	/	1/1	1/2	/	1/3	/	/	3/9
Total	8/26	18/25	22/23	30/47	0/2	8/20	12/25	3/4	101/172

Through quantitative statistical analysis of the data, we could see the numbers of sentences and inspirations retrieved by each participant. Participant 1 produced numerous sentences of memory retrieval and had the largest number of inspirations. Both sentences and inspirations cover almost all the categories. However, he/she had a poor conversion ratio in Category 6. Participant 3 provided the lowest number of sentences and inspirations. Participants 2, 3 and 5 preferred to retrieve events of product experience more than other participants. Their inspirations do mainly come from Category 4.

At the same time, from the total number of sentences and inspirations in each category, we could clearly see the contribution of different categories of retrieved memory to the idea generation. Category 4 have the largest number of sentences and inspirations, while Category 3 have the highest conversion ratio. The conversion ratio of Category 2 is higher than that of Category 1, while the domain distance of former from the topic is slightly far further than the latter. The Categories 5 has the least number of sentences. Although the number of sentences in Category 8 is also small, the conversion ratio is relatively high.

## Discussion

### The role of categorization in design theory

#### Using categorization as a research perspective

The designer’s self-memory is the most important information processor, which can affect the way designers judge the information they are exposed to, selectively absorb the categories they need, and potentially affect the perspective they use to analyze design problems. Categorizing the contents of memory retrieval is helpful for designers to recognize and optimize the knowledge structure. This study verified the eight categories identified by clustering and focus group analysis. And the verification studies confirmed the necessity of the existence of categories. The eight categories that are sufficiently detailed and valuable to be able to express complete information are a further complement to previous studies. At the same time, some categories of information that have been neglected by other studies have also shown their value on the conversion ratios that inspire ideas in subsequent studies.

The eight categories of memory retrieval are independent but interrelated. For example, the product-related experience in Category 4 and environmental analysis information in Category 6 are further supplementary to the memory content in Categories 1 and 2. Category 7 product analysis information often reflects the designer’s content of Category 8 design knowledge. As a designer, it is necessary to have a comprehensive and solid professional memory, while repetition and association of different categories of knowledge is undoubtedly a way to obtain long-term and effective memory. The categorization criteria proposed in this study can further improve the efficiency of designers’ knowledge association and defect detection.

#### Application of semantics and brainstorming method

Many studies on brainstorming have developed different approaches [51] and rules [52] to improve individual productivity and creativity. The common goal of these studies was to ensure that participants’ thoughts were not obstructed and increasing the number of ideas. However, The individual language expression is influenced by personal habit, context, social culture and other factors. The subjective judgements of linguistic meaning affected the rigor of analysis on the contents of brainstorming.This study proposed four criteria [Distance of descriptions to the topic, Description Type, Description Property, and Valence] in combination with semantic research [42]. Compared with categorizing sentences directly, the hierarchical clustering was a more efficient way in dealing with the scores of each sentence in the four criteria.

The eight categories proposed in this study can be used to analyze the brainstorming process that has been carried out. The categorized content can better reflect the diversity of thoughts and ideas. The distribution analysis in each category helps to evaluate and summarize the brainstorming content. In the future brainstorming activities with similar topics, it is possible to consciously control the brainstorming activities by adjusting the proportion of various information categories according to the categorization, so as to enrich the content of thinking.

### Application to design education

#### Understanding individual strength and weakness

Cognitive information processing theory points out that the deeper the information processing, the longer the memory retention [53]. Information can be reasonably categorized by categorization theory. Constant comparisons and analogies between information and existing concepts or knowledge can help establish relationships between knowledge. Combining the data shown in Table 4, each participant could identify and understand their strengths and weaknesses. This helps designers understand and reflect upon their performances of memory retrieval in design process and increase the conversion efficiency of ideas generated through memory retrieval. As for Participants 4 and 13, the frequency of non-similar objects they retrieved are more than that provided by other categories in design process, and so do inspirations in Table 4. It indicated that they want to be inspired from other domains, such as a sculpture design (Cluster 18) and a painting (Cluster 19) (S2 Data). After understanding the source of their inspiration, designers can be advised to moderately exercise the association between such information and the design topic to improve the retrieval efficiency.

#### Supposition on design fixation

Design fixation is a phenomenon that memory retrieval information has a negative influence on the design process. In the discussion of enhancing the memory retrieval of a certain category that helps design motivation, we can also avoid a certain category that hinders design motivation information. For instance, the differences among Category 1 (Congeneric product design), Category 2 (Similar product design) and Category 3 (Non-similar objects) are their distances to design topic. Similarly, in design fixation research field, researchers have studied the distances from stimuli to the concept of design issues [32–34]. However, the existing studies are more focused on stimulating innovation through the design of target transfer [54], few study regard events as stimuli while exploring design fixation. Therefore, based on existing theories of retrieval and categorization models [55, 56], further study is needed to help designers organize information in a favorable way to stimulate design and avoid design fixation.

## Conclusions

This study categorized retrieved memory exemplars based on designer students’ brainstorming on library desk and chair design. Through think aloud, we collected the sentences of describing memory content and tentatively categorized them into eight categories. Verification and Application study were carried out to prove the accuracy and value of this categorization.

Memory retrieval is an important stage of cognitive exploration in design activities. Categorizing the content of memory retrieval can help researchers further understand design thinking and extend the research field of design fixation. Students can also benefit from the categorization in realizing their individual design performances, through which teachers are able to partly evaluate students’ abilities. It is our future work to apply the categorization to other design education fields and design fixation.

Inevitably, there are some problems in our work. We only collect the sentences under the topic of library desk and chair design but have not verified the categorization procedure in other fields, which may restrict the application scope of this categorization. In addition, the number of participants in sentence collection should be increased to strengthen the reliability of our analysis results.

## Supporting information

S1 Data 172 sentences. Two recorders recorded 172 sentences spoken by participants in the brainstorming experiment.
(XLSX)

S2 Data 152 sentences. 152 sentences were preserved by combining similar sentences in 172 sentences without affecting the meaning of the sentence description. Typical sentences of 24 clusters were bolded and marked.
(XLSX)

S3 Data Mean value of four criteria of 152 sentences. The mean value was obtained by thirty-one college students who scored 152 sentences according to four categorization criteria (Distance to the topic, Description type, Description property, Valence) through a questionnaire.
(XLSX)

S1 Table Categorization verification questionnaire (No.52 as example). (DOCX)
