# Supplementary material for: Content categorization for memory retrieval: A method for evaluating design performance
Source: PLoS One. 2023 Jan 19;18(1):e0280459. doi: 10.1371/journal.pone.0280459 (PMC9851509; doi:10.1371/journal.pone.0280459)
Supplement: S1 Table — (DOCX) [file pone.0280459.s004.docx]

**S4 Table. Categorization verification questionnaire (No.52 as example).**

| Case 6 There is a sofa I have seen before which books can be plugged into. | |
| --- | --- |
| A1 Do you think which category the sentence belongs to? | A2 Do you think which category the sentence belongs to? |
| 1. Existing library table and chair design  2. Other types of table and chair design  3. Objects unrelated with library table and chair  4. Event associated with table or chair in library  5. Event irrelevant with table or chair in non-library  6. Library analysis information  7. Table and chair analysis information  8. Design research and methods  9. Others | 1. Congeneric product design  2. Similar product design  3. Non-similar objects  4. Product-related experience  5. Product unrelated event  6. Environment analysis information  7. Product analysis information  8. Design knowledge  9. Others |
|  |  |
